# Effect of practice on learning a balance task in children, adolescents, and young adults

**DOI:** 10.3389/fpsyg.2022.989645

**Published:** 2022-10-06

**Authors:** Thomas Muehlbauer, Dennis Brueckner, Simon Schedler

**Affiliations:** Division of Movement and Training Sciences/Biomechanics of Sport, University of Duisburg-Essen, Essen, Germany

**Keywords:** postural control, skill acquisition, retention, transfer, growth, development, age

## Abstract

**Background:**

A lower developmental stage of the postural control system in childhood compared to adolescence and adulthood was reported in numerous studies and suggests differences (i.e., less improvements in children than in adolescents and young adults due to the immature postural control system) during learning a balance task. Therefore, the present study examined the effect practice on learning (i.e., retention and transfer) a balance task in healthy children, adolescents, and young adults.

**Methods:**

Healthy children (*n* = 32, 8.5 ± 0.5 years), adolescents (*n* = 30, 14.6 ± 0.6 years), and young adults (*n* = 28, 24.3 ± 3.3 years) practiced balancing on a stabilometer (i.e., to keep the platform as close to horizontal as possible) for 2 days. On the third day, learning was assessed using a retention (i.e., balance task only) and a transfer (i.e., balance task plus concurrent motor interference task) test. The root-mean-square-error (RMSE) was calculated and used as outcome measures.

**Results:**

Over the course of practice, significant improvements (*p* < 0.001) were detected in favor of children and young adults. However, neither the retention nor the transfer test showed significant group differences.

**Conclusion:**

Our findings indicate that learning a balance task did not seem to be influenced by the developmental stage of the postural control system.

## Introduction

A well-developed postural control system is important for the successful execution of everyday (e.g., climbing stairs) and sports-related (e.g., balancing on a beam) activities. The development of postural control is characterized by children showing faster sway (Hytonen et al., 1993), lower walking speeds (McKay et al., 2017b), and smaller reach distances (McKay et al., 2017a) than adolescents, who in turn show worse values compared to young adults. Ongoing growth and maturation processes are reported as causes that are approximately completed at the transition from adolescence to adulthood (Hirabayashi and Iwasaki, 1995). Precisely, it has been shown that the ability to use somatosensory input matures rather early at the age of 3–4 years, while it is not before adolescence (i.e., 15–16 years) that visual and vestibular function reach their full potential (Hirabayashi and Iwasaki, 1995; Steindl et al., 2006).

Besides these processes, improvements in postural control can be achieved at all ages through physical practice and training (Lesinski et al., 2015; Gebel et al., 2018). For example, Schedler et al. (2020b) reported significantly improved balance performance in children (mean age: 8.5 ± 0.5 years) following 2 days of practice on a stability platform (stabilometer). In addition, a study with adolescents (mean age: ~12 years) showed improvements in stance duration (i.e., one-legged stance) and reach distances (i.e., Y balance test) after 7 weeks of balance training (Schedler et al., 2020c). Lastly, Gruber et al. (2007) found significantly reduced postural sway in young adults (mean age: ~26 years) after 4 weeks of balance training. Despite the associated gain in knowledge, no age-specific differences in the effectiveness of balance practice/training can be deduced, as these are individual studies that used different experimental procedures (i.e., balance tests/outcomes, practice/training loads). In other words, it remains unclear whether the positive influence of balance practice/training on balance performance is affected by the age-related development of the postural control system. However, in addition to developmental processes, there is evidence of practice- and age-related increases in knowledge and experience that have a positive influence on balance control. For example, Garcia et al. (2011) reported smaller sway area and less sway velocity during quiet upright stance in gymnasts (9–11 years) compared to age-/sex-matched controls. Further, Busquets et al. (2018) showed shorter postural sway and less time for balance recovery during quiet standing in an adolescent (≥15 years) versus child (8–11 years) cohort of gymnasts.

Thus, the purpose of the present study was to directly compare the effect of practice on learning a balance task in children, adolescents, and young adults within one study using the same methodology across age groups. We assumed that practice leads to improvements in balance performance, which would be greater in young adults (due to the fully matured postural control system) compared to children and adolescents. Further, we hypothesized that practice would induce learning (i.e., retention and transfer) with young adults showing better balance performance than children and adolescents.

## Materials and methods

### Participants

Thirty-two children, 30 adolescents, and 28 young adults participated in the study (Table 1). All participants were healthy and free of any neurological or musculoskeletal impairments. None of the participants had prior experience with the balance task. Written informed consent and subject’s assent were obtained from all participants before the start of the study. Additionally, parent’s approval was obtained for the children and adolescents. The Human Ethics Committee at the University of Duisburg-Essen, Faculty of Educational Sciences approved the study protocol (approval number: TM_10.07.17).

**Table 1:** Characteristics of the study participants by group.

Characteristic	Children (*n* = 32)	Adolescents (*n* = 30)	Young adults (*n* = 28)	Value of *p*
Age (years)	8.5 ± 0.5	14.6 ± 0.6	24.3 ± 3.3	<0.001
Gender (f, m)	16, 16	15, 15	14, 14	–
Body mass (kg)	32.5 ± 5.9	60.0 ± 10.0	76.5 ± 1.2	<0.001
Body height (cm)	137.8 ± 6.6	168.1 ± 8.2	177.0 ± 8.5	<0.001

Data represent means ± standard deviations. f, female; m, male.

### Experimental procedure

**Figure 1:**
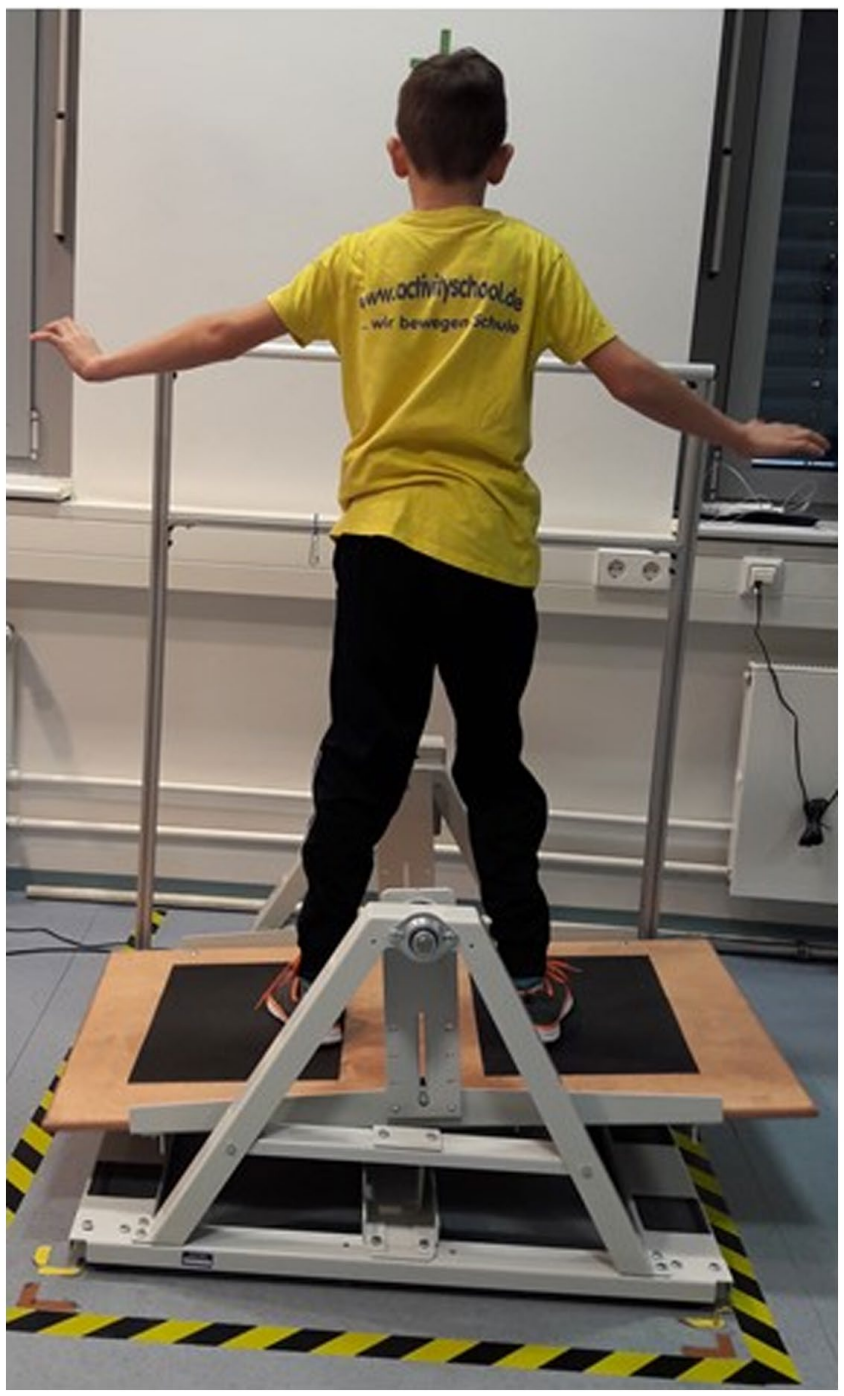
Illustration of a participants balancing on the stability platform (stabilometer). Participant’s assent and parents’ written informed consent were obtained to publish this figure.

The study took place on three consecutive days. On all days, participants were instructed to balance with eyes opened and while wearing shoes on a stability platform (i.e., to keep the platform horizontal ±3°) that was equipped with a safety rail and consisted of a swinging wooden platform (65 × 107 cm) which allowed a maximum deviation of 15° to either side of the horizontal plane of the platform (Figure 1). On day 1 and day 2, seven practice trials each lasting 90 s were performed. The inter-trial rest period amounted to 90 s. Each trial started from the horizontal position with participants holding on to the safety rail. During practice, the subjects were allowed to grab the safety rail if there was a risk of losing balance. After each trial, participants stepped off the platform and received knowledge of results (KR) using “time in balance” (i.e., when the platform angle was within ±3° of the horizontal position).

On day 3, retention and transfer tests were carried out to assess learning of the balance task. The procedure during retention test was the same as for the acquisition phase, yet participants performed only three trials á 90 s and did not receive KR as described in previous studies (Maxwell and Masters, 2002; Wulf et al., 2003; Jackson and Holmes, 2011). Following a rest period of 90 s, the transfer test took place (3 trials á 90 s), which involved the execution of an additional motor interference task (Laessoe et al., 2008). Specifically, the participants were instructed to hold two connected metal rings in a way that they did not touch each other while balancing on the platform for another three trials. For the transfer test, no KR was given as well.

### Data collection

The stability platform was equipped with an angle transducer (sampling rate: 25 Hz) measuring the platform position over the entire trial duration of 90 s using PsymLab software (Lafayette, LA, United States). For data analysis, the root mean square error (RMSE) of the angle data (degree) in relation to the horizontal platform position (corresponds to 0°) was calculated over the 90-s trial duration and used for further statistical analysis.

### Statistical analysis

Descriptive statistics were presented as means ± standard deviations. Normal distribution was examined using the Shapiro–Wilk test and homogeneity of variances using the Levene test. A univariate analysis of variance (ANOVA) was used to detect baseline-differences (i.e., 1st trial at day 1) between groups. Further, a 3 (Group: children, adolescents, young adults) × 2 (Day: 1–2) × 7 (Trial: 1–7) ANOVA with repeated measures on Day and Trial was performed to assess group discrepancies during the acquisition phase. Lastly, a 3 (Group: children, adolescents, young adults) × 2 (Test: retention, transfer) ANOVA with repeated measures on Test was used to detect group-specific learning effects. All analyses were performed using the SPSS (version 27.0) and the significance level was set at *p* < 0.05.

## Results

**Figure 2:**
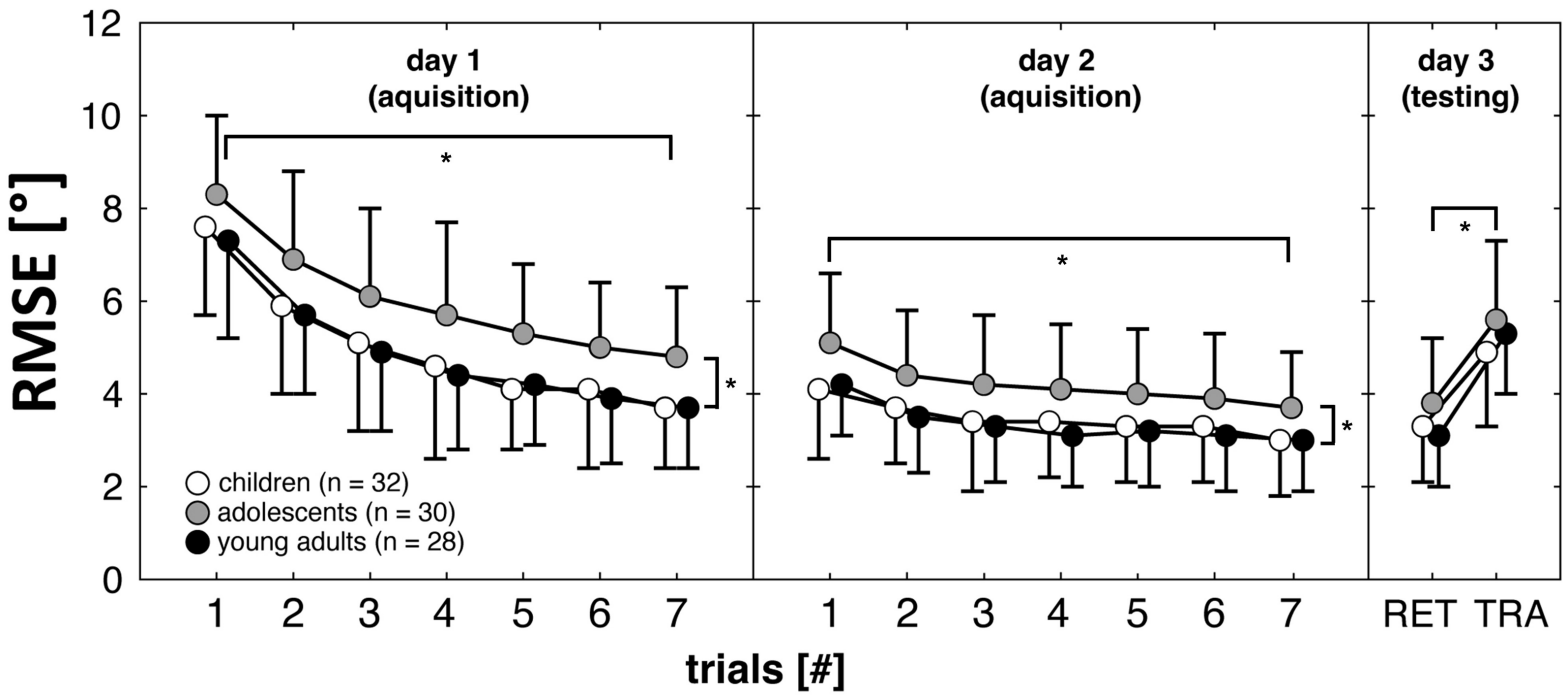
Root mean square error (RMSE) in degree for the children (unfilled circles), adolescents (grey filled circles), and young adults (black filled circles) during acquisition (day 1 and day 2) and during testing (day 3). Data represent means and standard deviations. ^*^Represents a statistically significant difference (*p* < 0.05). RET, retention test (i.e., balance task only); TRA, transfer test (i.e., balance task plus concurrent motor interference task).

Group-specific changes of the RMSE (degree) during acquisition, retention, and transfer are displayed in Figure 2. There were no baseline-differences (*F*_(2, 89)_ = 2.279, *p* = 0.108) between groups. During acquisition on day 1 and 2, the Group × Day × Trial ANOVA revealed main effects of Day (*F*_(1, 87)_ = 402.266, *p* < 0.001), Trial (*F*_(6, 522)_ = 237.075, *p* < 0.001), and Group (*F*_(2, 87)_ = 5.332, *p* = 0.007). Thus, all groups (adolescents less than children and young adults) reduced their RMSE values across practice. Yet, we did not detect a significant Group × Day × Trial interaction (*F*_(12, 522)_ = 0.423, *p* = 0.954). During testing on day 3, the Group × Test ANOVA yielded a main effect of Test (*F*_(1, 87)_ = 317.456, *p* < 0.001) but not of Group (*F*_(2, 87)_ = 1.790, *p* = 0.173). Thus, all groups performed better in the retention than in the transfer test. Further, the Group × Test interaction (*F*_(2, 87)_ = 2.576, *p* = 0.082) did not reach the level of significance, indicating that learning was not age-specific.

## Discussion

Partially in line with our first hypothesis, we observed balance improvements during practice, that were larger not only in young adults but also in children compared to adolescents. In accordance, previous studies (Wulf et al., 1998; Jackson and Holmes, 2011) using the stabilometer device also found enhancements in balance performance following 2 days of practice. Young adults possess a fully matured postural control system (Shumway-Cook and Woollacott, 1985), which explains their larger enhancements compared to adolescents. However, children compared to adolescents also showed greater improvements although both have a still maturing postural control system (Nolan et al., 2005). In this regard, using the sensory organization test Hirabayashi and Iwasaki (1995) and Steindl et al. (2006) revealed that some aspects of postural control, especially vestibular function do not even reach the adult-level before the age of 15 years. Greater training-related balance improvements in children compared to adolescents were also reported by Walchli et al. (2018) and Schedler et al. (2020a). From these results, the authors concluded the existence of an adaptive reserve regarding balance controlling structures (e.g., central nervous system, sensorimotor function). These structures mature throughout childhood and adolescence into adulthood (Shumway-Cook and Woollacott, 1985), whereby the adaptive potential seems to be greater in children than in adolescents. In addition, adolescence compared to childhood is characterized by growth spurts (i.e., uneven growth processes; Tanner and Whitehouse, 1982). These can lead to motorically awkward movements (Butterfield, 2015), which could further explain the lower balance improvements in adolescents than in children.

Contrary to our second hypothesis, no significant age differences during testing neither for the retention nor for the transfer condition were found. Therefore, the presence of a fully developed postural control system in adults versus the presence of an adaptive reserve in children and adolescents does not appear to differentially affect the learning of a balance task. From a practitioner’s perspective, the results indicate that despite differences in the developmental stage of the postural control system, practicing a balance task leads to similar improvements in balance in all of the investigated age groups. Therefore, practicing and training of balance should be performed regardless of participant’s age.

## Conclusion

Practicing a balance task resulted in significantly improved balance performance in children, adolescents, and young adults, with smaller enhancements in adolescents. However, no significant age differences were revealed in the retention and transfer test, indicating equal learning regardless of the developmental stage of the postural control system. This implies that although growth, development, and maturation seem to affect improvements during practice of a novel balance task to some degree, they appear to play a minor role in learning (i.e., retention and transfer test) a balance task.

## Limitations and future research

The present study has some limitations that should be addressed in future research. First, the detected effects refer to short-term practice over several days which limits the transfer to longer-lasting practice periods. Thus, future studies should apply mid-term (i.e., several weeks) and long-term (i.e., several months) practice periods to see whether age differences occur with respect to learning a balance task. Second, the used balance task (i.e., balancing on a stabilometer) is rather artificial, which limits transferability of the findings to everyday or sports-related balance activities. Consequently, further studies should investigate whether age differences are more likely to be evident in recreational (e.g., balancing on a slackline) or sports-related (e.g., walking on a balance beam) tasks with balance demands. Third, only a behavioral measure (i.e., RMSE) was calculated, leaving the underlying neuromuscular adaptations unclear. Therefore, additional neuronal (i.e., functional and structural brain changes) and muscular (i.e., muscle activity) correlates should be investigated in the future. Fourth, in order to achieve a stable stance on the platform, the balancing task was performed while wearing shoes. Therefore, sensory information could only be used to a limited extent for balance control.

## Data availability statement

The raw data supporting the conclusions of this article will be made available by the authors, without undue reservation.

## Ethics statement

The studies involving human participants were reviewed and approved by Human Ethics Committee at the University of Duisburg-Essen, Faculty of Educational Sciences. Written informed consent to participate in this study was provided by the participants’ legal guardian/next of kin.

## Author contributions

TM and SS designed the study and wrote the main parts of the manuscript. DB and SS planned and supervised the practice and testing phases. DB conducted the data collection. TM analyzed the data. All authors contributed to the article and approved the submitted version.

## Funding

We acknowledge support by the Open Access Publication Fund of the University of Duisburg-Essen. The funding body is independent of the design of the study and collection, analysis, and interpretation of data and in writing the manuscript. Open access funding is enabled and organized by the project DEAL.

## Conflict of interest

The authors declare that the research was conducted in the absence of any commercial or financial relationships that could be construed as a potential conflict of interest.

## Publisher’s note

All claims expressed in this article are solely those of the authors and do not necessarily represent those of their affiliated organizations, or those of the publisher, the editors and the reviewers. Any product that may be evaluated in this article, or claim that may be made by its manufacturer, is not guaranteed or endorsed by the publisher.
